# Supplementary figures and images for: The Drosophila amyloid precursor protein homologue mediates neuronal survival and neuroglial interactions
Source: PLoS Biol. 2020 Dec 8;18(12):e3000703. doi: 10.1371/journal.pbio.3000703 (PMC7723294; doi:10.1371/journal.pbio.3000703)

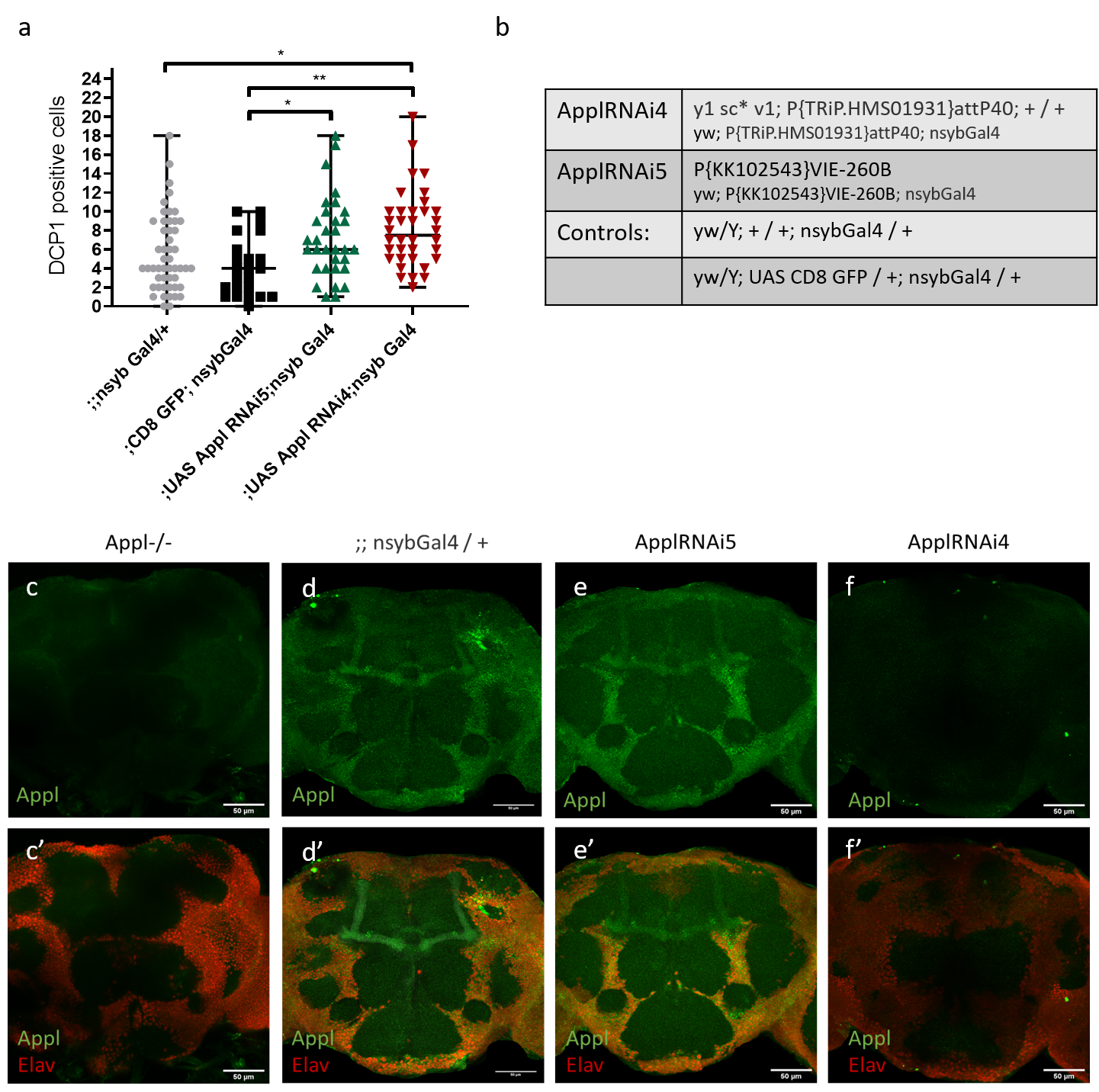

Supplement: S1 Fig — (a) This graph shows the number of apoptotic cells in the central brain of control,;; nsyb Gal4 and; UAS CD8 GFP; nsyb Gal4, and 2 different Appl RNAi flies at 7 days old. Each data point represents the number of apoptotic cells, Dcp-1 positive cells, in a single brain. For the analysis of these data, we used 1-way ANOVA with Tukey multiple comparison test F(3,137) = 6.050, df = 3, *p = 0.0176, *p = 0.0252, **p = 0.0012. (b) This table describes the genotype of the 2 different RNAi lines used and the controls. (c–f’) Confocal images of the central brain of each genotype stained with anti-App (green) to observe the expression levels of APPL. Underlying data can be found in the S1 Data file. (TIF) [file pbio.3000703.s001.tif]

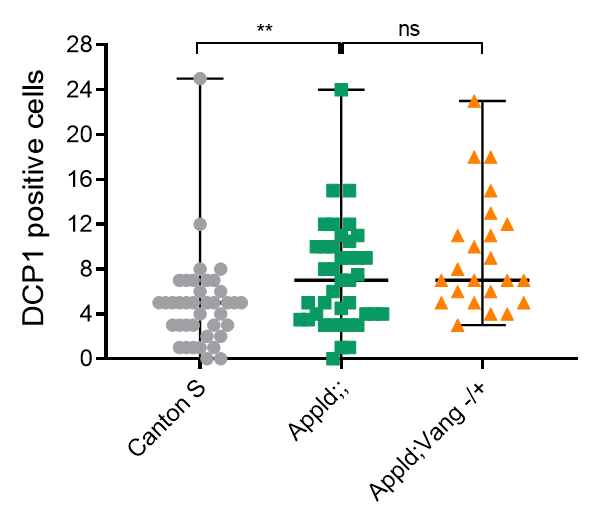

Supplement: S2 Fig — Quantification of apoptotic cells in the central brain of control, w*/Y;+/+;+/+, w*appld/Y;;, and appldw*/Y;Vang-/+;. Reducing 1 copy of Vang, a key member of the Wnt PCP pathway, in an APPL-/- background has no effect on the accumulation of apoptotic cells. Underlying data can be found in the S1 Data file. (TIF) [file pbio.3000703.s002.tif]

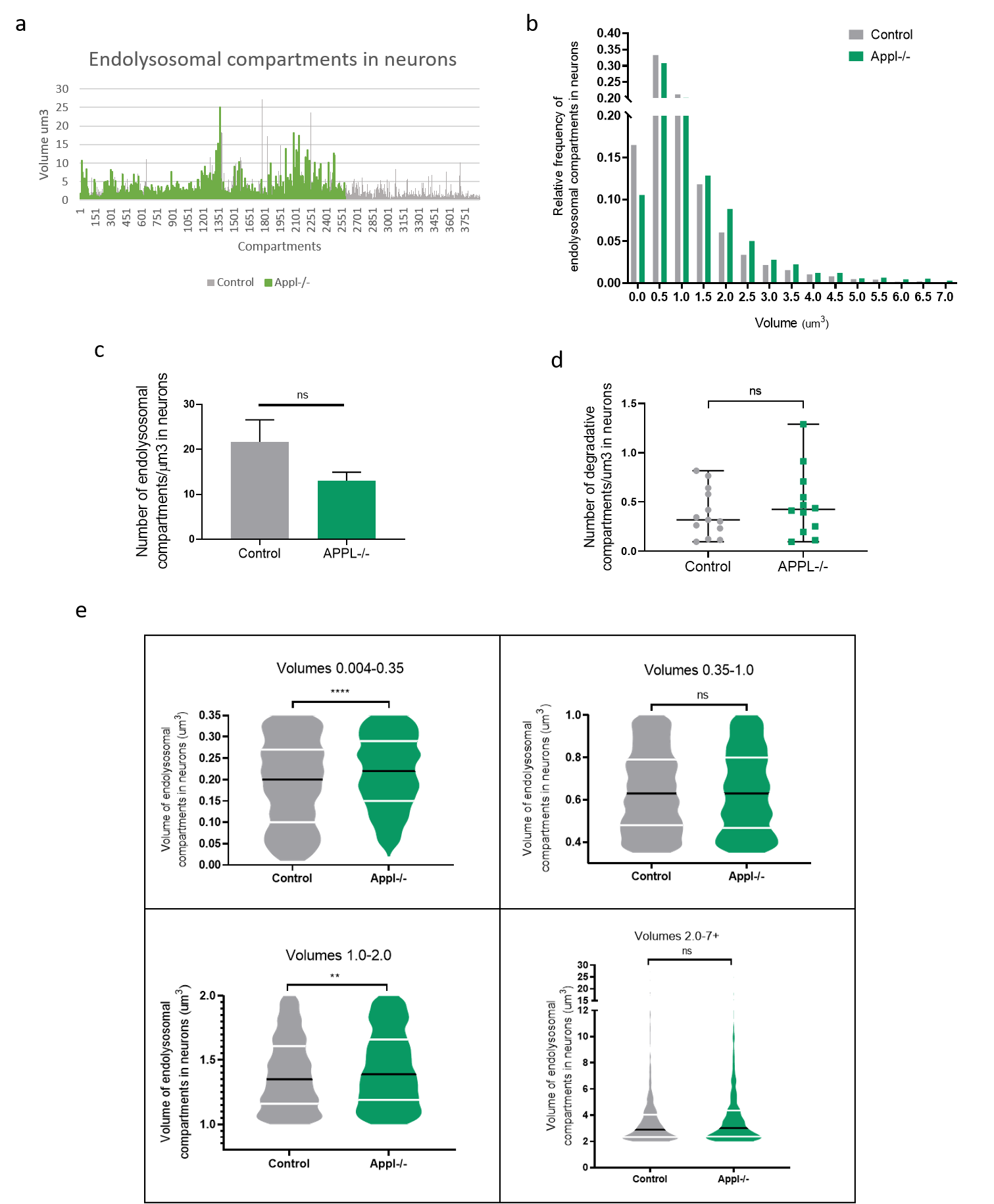

Supplement: S3 Fig — (a) Histogram presenting the volume of each endolysosomal compartment in neurons of control, w*;UAS myr mCherry pH Luorin; nsyb Gal4 fly, and appld mutant: APPLd; UAS myr mCherry pH Luorin; nsybGal4, flies. (b) This histogram presents the relative frequency of endolysosomal compartments in neurons. (c) This graph shows the quantification of the number of endolysosomal compartments/um3 in neurons, which is not significantly different between control and Appl-/- flies. (d) This graph shows that the number of degradative compartments/um3 is also not significantly affected by the absence of APPL, every dot corresponds to a brain. (e) These 4 panels represent the same data as in Fig 2D but separated in smaller groups of volume ranges; endolysosomal compartments of 0.004 to 0.35 um3, 0.35 to 1 um3, 1 to 2um3, and 2 to 7 um3. As observed, the most important differences in size are in the smallest volume group ****p < 0.0001 and the 1 from 1 to 2 um3, **p = 0.003, Mann–Whitney test. Underlying data can be found in the S1 Data file. (TIF) [file pbio.3000703.s003.tif]

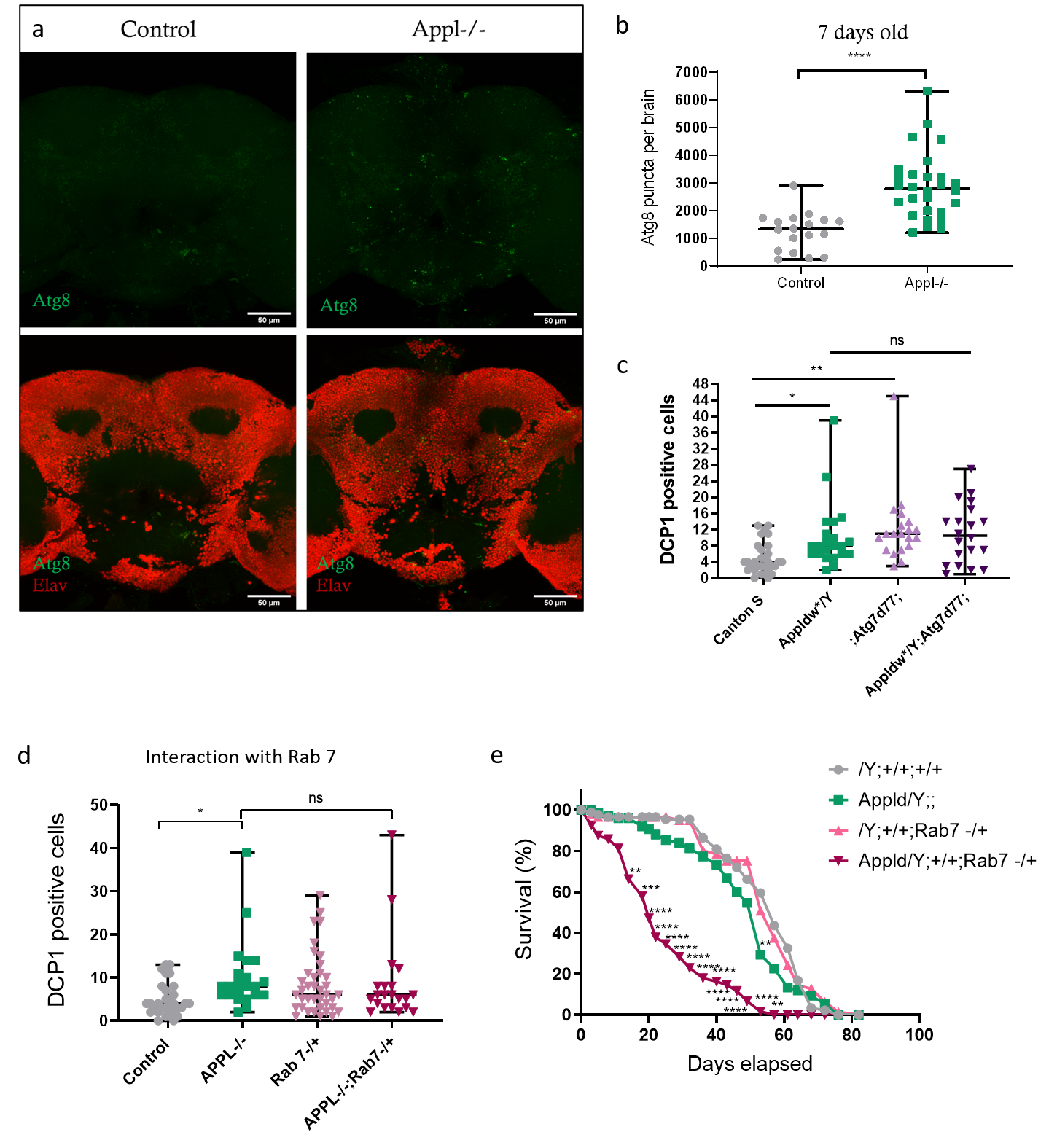

Supplement: S4 Fig — (a) Confocal stack of the central brain stained with Elav (red) to mark the neuronal cell bodies and Atg8 (green) to mark the autophagosomes. In this picture, we compare Canton S (control) flies to Appl-/-. (b) Graph presenting the increased number of Atg8 puncta per brain in appld background flies at 7 of age. Mann–Whitney test ****p < 0.0001. (c) This graph represents the quantification of apoptotic cells in the brain of appld background flies with 1 reduced copy of the Atg7 gene. (d) Graph presenting the quantification of Dcp-1 positive cells in the brain of 7-day-old APPL-/- flies lacking 1 copy of Rab7, the late endosome marker, w*appld/Y;;Rab7 KO Crispr 3P3RFP/+, compared to control and Appl-/- flies. This graph shows no difference in the number of apoptotic neuronal cell death when 1 copy of Rab7 is reduced. (e) Life span analysis of control and appld flies lacking 1 copy of Rab7. This survival curve reveals that reducing 1 copy of Rab7 in an appld background increases significantly the death rate of Appl-/- flies, starting from an even earlier age and reducing the overall life span of appld flies, 2-way ANOVA with Tukey multiple comparison test **p = 0.0022, ****p < 0.0001. Underlying data can be found in the S1 Data file. (TIF) [file pbio.3000703.s004.tif]

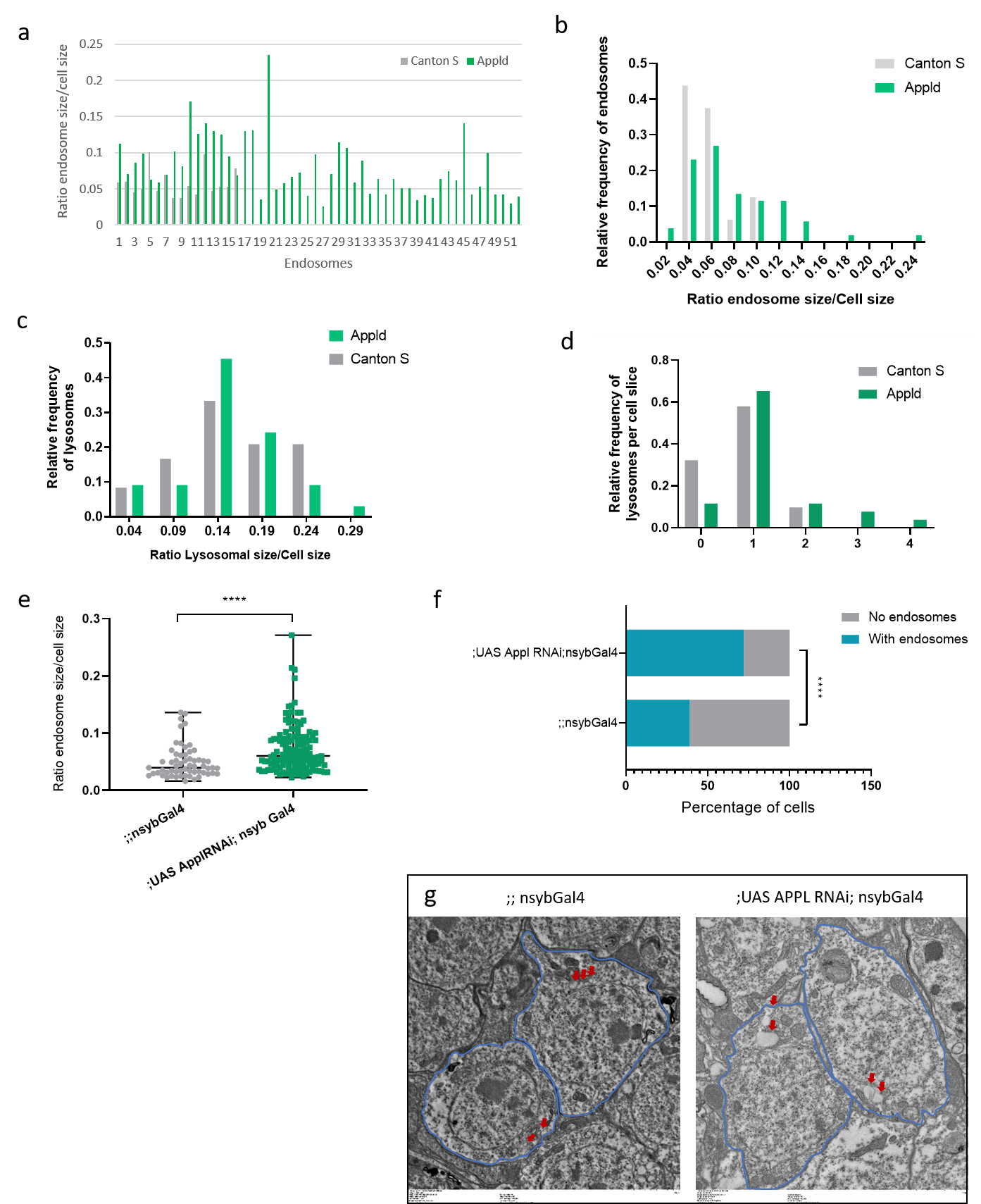

Supplement: S5 Fig — (a) Histogram presenting the volume of each early endosome–like vesicle in neurons of control, +/+;+/+;+/+ and APPL-/- flies, w*appld/Y;; flies. (b) This histogram represents the relative frequency of early endosome–like vesicles and their size in APPL-/- flies, w*appld/Y;;, comparing to control, +/+;+/+;+/+. c) This histogram represents the relative frequency of lysosomes and their size in APPL-/- flies, w*appld/Y;;, comparing to control, +/+;+/+;+/+. (d) Histogram representing the relative frequency of lysosomes per cell slice in APPL-/- flies, w*appld/Y;;, comparing to control, +/+;+/+;+/+ fly brains. (e) Focusing on the 7-day-old time point, which showed significantly enlarged early endosome–like vacuoles in Appl-/- flies (Fig 3d), we now knock down the expression of APPL only in neurons using the yw; UAS APPL RNAi (y+); nsybGal4 and find a similar increase in the size of these early endosome–like vacuoles comparing to the control: yw;;Gal4nsyb/+. n = 3 to 5 brains per genotype and approximately 60 cells analyzed per genotype, ****p < 0.0001. (f) As in (Fig 3E), the percentage of cells with endosomes was significantly higher in yw; UAS APPL RNAi (y+); nsybGal4 comparing to control. n = 3 to 5 brains per genotype and approximately 60 cells analyzed per genotype, Fisher exact test ****p < 0.0001. (g) TEM horizontal sections of the cortical region of 7-day-old fly brains showing neuronal cell bodies (circled in blue) and the early endosome–like vacuoles marked with a red arrow. n = 3 to 5 brains per genotype and approximately 60 cells analyzed per genotype. Underlying data can be found in the S1 Data file. (TIF) [file pbio.3000703.s005.tif]

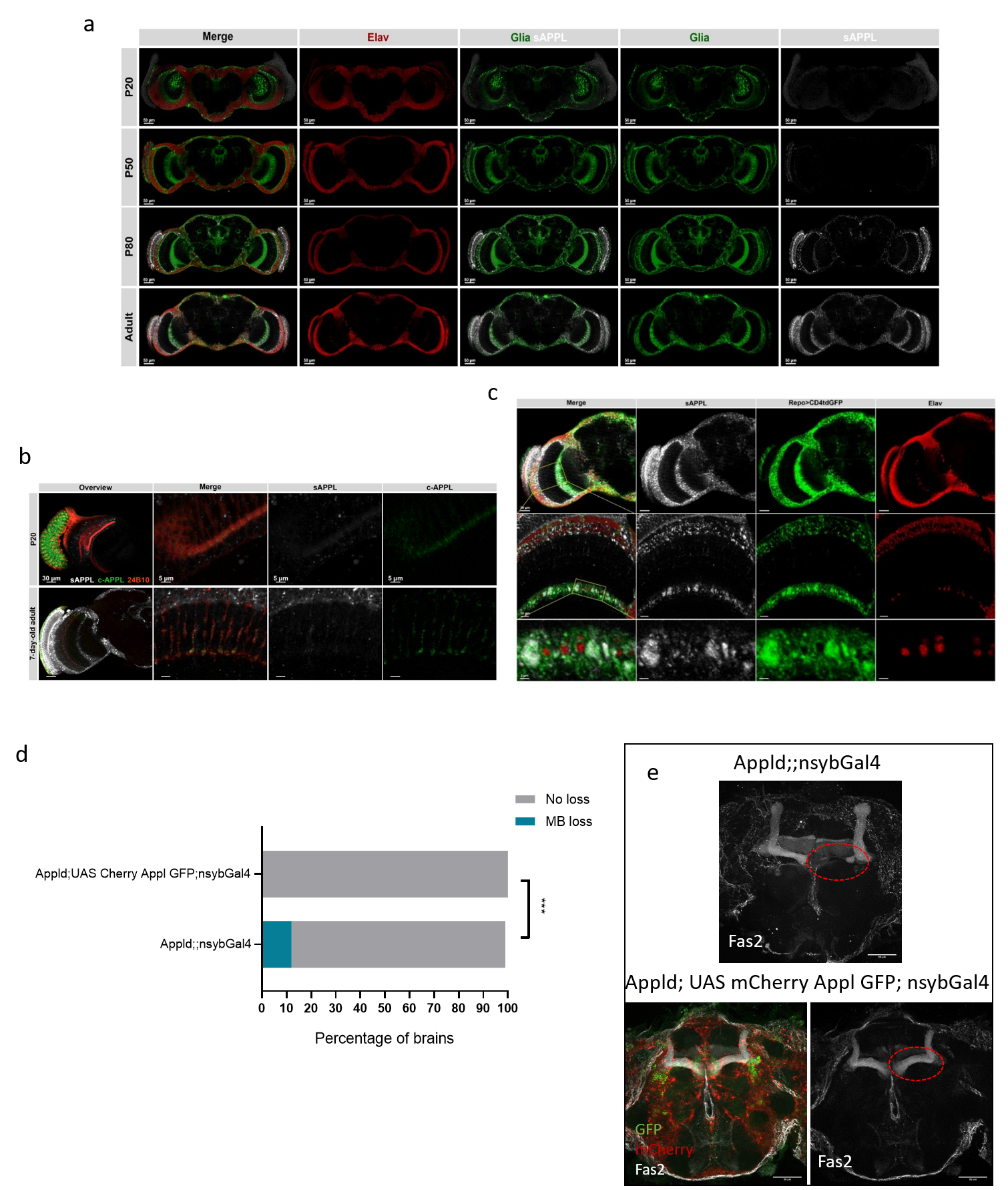

Supplement: S6 Fig — (a) Confocal sections of a control fly brain throughout development until adulthood that expresses the double fluorescent tagged APPL construct specifically in the retina using the GMR Gal4 driver,; UAS-mCherry-APPL-GFP/lexAop-CD4tdGFP; GMR-Gal4/Repo-lexA. As we can see between P50 and P80, there is a significant release of SAPPL (white) beyond the site of expression reaching all areas of the brain. (b) This is a different experiment using these flies:; UAS-mCherry-APPL-GFP; GMR-Gal4. These close-ups on the photoreceptors confirm that it is only the SAPPL (white) that travels ubiquitously in the brain, although the carboxyl terminus of APPL (green), the intracellular part, remains in the cell bodies where it is being expressed. (c) This graph shows the adult stage of the flies used in (a) and highlights that the SAPPL (white) not only travels throughout the brain but also colocalizes specifically with the glial marker, Repo (green). (d and e) Rescue experiment of the loss of mushroom body (MB) β lobes (red oval) phenotype in Appld;;nsybGal4 flies with a 12.2% phenotype penetrance (n = 49). Appld; UAS mCherry Appl GFP; nsybGal4 is functional as it rescues this loss of MB β lobes with a 0% phenotype penetrance (n = 39). ***p = 0.0002 Fisher exact test. Underlying data can be found in the S1 Data file. (TIF) [file pbio.3000703.s006.tif]

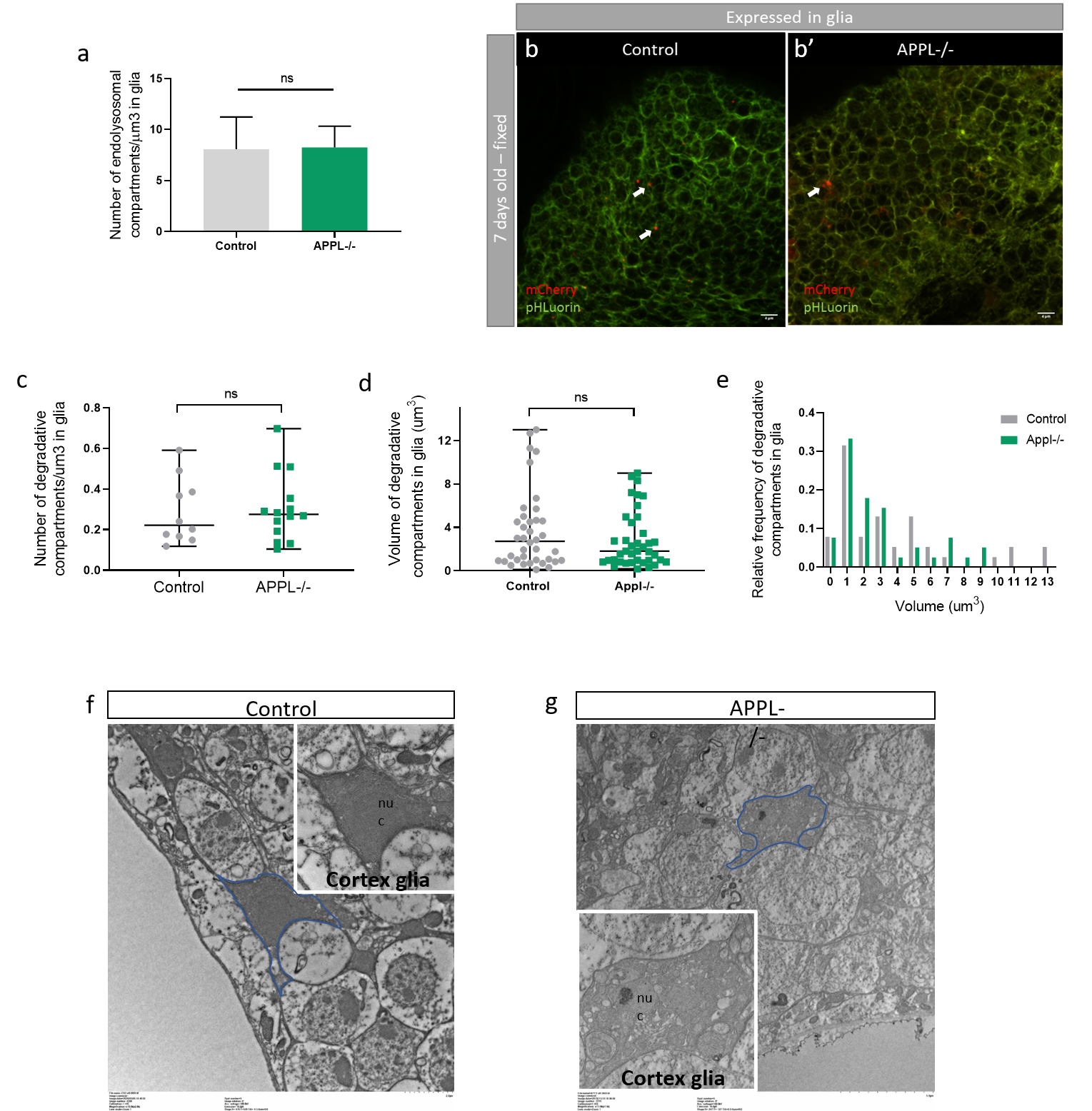

Supplement: S7 Fig — (a, c) The number of endolysosomal and degradative/acidic compartments were not affected by the absence of APPL in glia. (b and b’) These confocal slices represent the same area of glial cells but this time from a fixed tissue of control and APPL-/- 7-day-old fly brains. (d and e) The volume of degradative/acidic compartments was also similar between both conditions. (f and g) TEM horizontal sections of the cortical region of a 7-day-old fly brain showing neuronal cell bodies and cortical glia (circled in blue) between them. We can observe that the distribution of cortical glia in the brain of APPL-/- flies is abnormal, they have an irregular shape and many vesicles, comparing to the control. Underlying data can be found in the S1 Data file. (TIF) [file pbio.3000703.s007.tif]

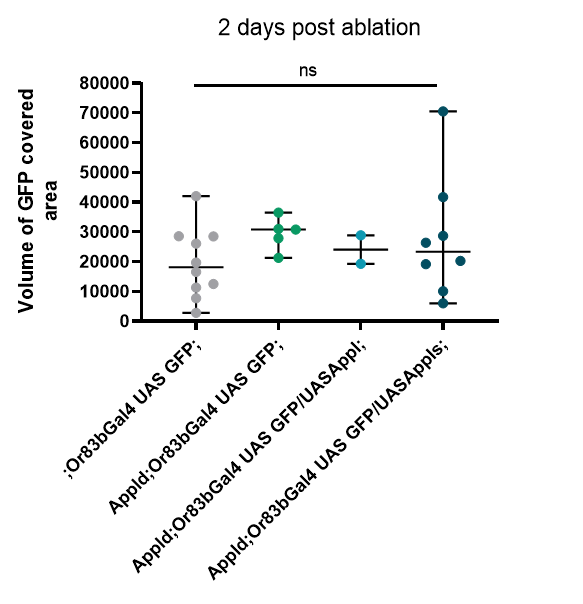

Supplement: S8 Fig — Quantification of volume of GFP-covered region (um3) in the OR83b innervating glomeruli at 2 days post-ablation, in control, APPL-/- and the rescue flies: Appldw*; UAS APPL/OR83bGal4GFP; and Appldw*; UAS SAPPL/OR83bGal4GFP;. Underlying data can be found in the S1 Data file. (TIF) [file pbio.3000703.s008.tif]

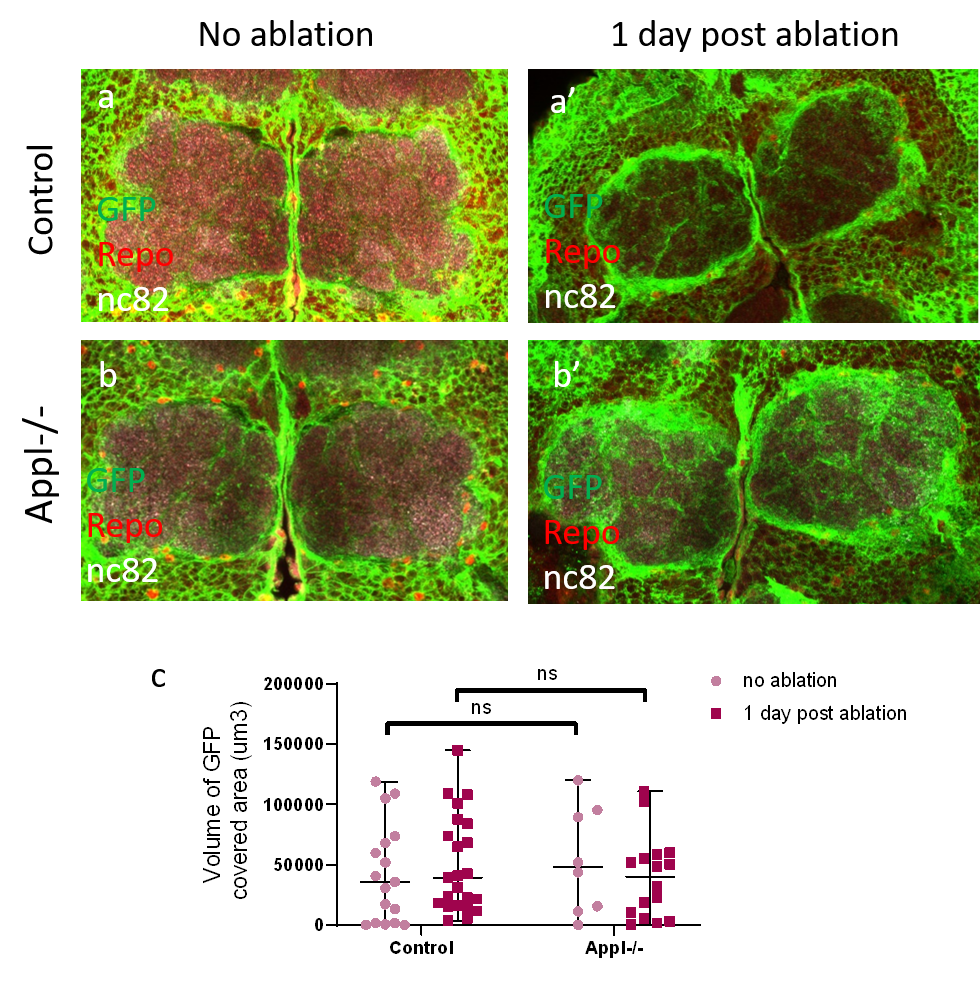

Supplement: S9 Fig — (a and a’) Confocal images of a control brain; UAS GFP/+;Repo Gal4/+ before and 1 day after ablation. The endogenous expression of GFP represents the glial migration around and inside the antennal lobes after antennal ablation. (b and b’) In Appl null flies Appld;UAS GFP/+;Repo Gal4/+, we observe a similar reaction of glial cells after antennal ablation. (c) This graph presents the quantification of the volume (um3) of GFP-covered area in and around the antennal lobes before and 1 day after ablation in control compared to Appl-/- flies. Underlying data can be found in the S1 Data file. (TIF) [file pbio.3000703.s009.tif]
