# Supplementary material for: The Drosophila amyloid precursor protein homologue mediates neuronal survival and neuroglial interactions
Source: PLoS Biol. 2020 Dec 8;18(12):e3000703. doi: 10.1371/journal.pbio.3000703 (PMC7723294; doi:10.1371/journal.pbio.3000703)
Supplement: Attachment — Submitted filename: Kessissoglou et al_ReviewerResponse.docx [file pbio.3000703.s012.docx]

**Editor’s comments:**

*Two reviewers seem to be concerned about the effect sizes, which are small, particularly on cell death and on endolysosomal volumes.*

We understand your concern on the effect sizes and we increased the n number wherever the reviewers mentioned it.

*We think that reviewer 3’s suggestions are quite appropriate. Please do the additional controls, such as rescue of the glial disruptions and endolysosomal phenotypes. This, we think, could eliminate the major concern that subtle differences in genetic background are at play here. In general, reviewer 3’s comments are sharp, we think, and you should address them thoroughly, including increasing the sample size in some experiments.*

We have performed several new experiments that we hope will eliminate the major concern that subtle differences in genetic background are at play here. For example, the new rescue experiment of the endolysosomal phenotype using TEM in Fig.4, the rescue experiment of glial activity, that we had already included in Fig.8, and the new experiments on the effect of loss of APPL on glial activity in Fig.9 and Fig.S10.

*You should show higher magnification images for figure 3, as you have done elsewhere.*

This point has been addressed and the Figure 3i was modified. It is now on Figure 5a.

*Reviewer 1 also raises a series of critiques that we think have more to do with interpretation of the data. We think that these sorts of critiques could be addressed with a more nuanced discussion of the data.*

We have addressed this issue by toning down the interpretation of our data accordingly.

*The Academic Editor also thinks, without wanting to be too instructive, that you could use the CPV reporter to mark apoptotic cells and glial cells. This is a UAS-driven construct that has reporters that are activated by caspase cleavage. I am passing this recommendation to you, in case you find it useful.*

This was a very good suggestion but, unfortunately, we received the CPV reporter flies only after the Covid-19 confinement and they were not healthy. We did not have the time to request the flies again and hence, we didn’t have time to do this specific experiment.

**Reviewer 1:**

***General comment:***

*The manuscript by Kessissoglou et al. aims at finding a physiological function of APPL in the adult Drosophila brain. The authors analyze appl null mutant and present here different aspects of its phenotype, such as lifespan, cell death in the adult brain, dimensions of endosomal and degradative compartments in neurons and glia and evaluation of glial capacity to clear injured axons.* ***The described results are interesting but too preliminary for making the presented conclusions.*** *The authors show that appl mutant flies exhibit a significantly reduced lifespan and accumulation of Dcp-1-positive cells in the adult brain as compared to control flies.* ***However, the presented figures of the anti-Dcp-1 staining are not convincing and the differences between control and mutant flies are based on an extremely low number of apoptotic cells in the adult brain (2-8).*** *In addition, this staining often shows a strong background like in Fig. 3i, which therefore cannot be used as the solitary detection and quantification of dying cells.* ***Moreover, accumulation of apoptotic cells often results from their impaired clearance and not necessarily from the excessive cell death. It is not clear from the presented data whether excessive neuronal death actually occurs in the appl mutant.*** *Based on the assessment of the volume of endosomal and degradative compartments in neurons, the authors claim their enlargement in the appl mutant brains compared to control. However,* ***the live imaging pictures are not of sufficient quality to quantify fluorescence and the number of analyzed brains is very low (2).*** *It is not clear to me whether and how the slightly increased volume of endosomal compartments leads to neuronal cell death.* ***The reduced capacity of glia to clear degenerating axons in the appl mutant, which is rescued by the full length APPL or SAPPL is an interesting result****. However, it does not certainly indicate the impaired glial ability to clear apoptotic neurons in the appl mutant. These are two distinct processes, which share some common mechanisms.*

We understand the concerns of Reviewer 1 and have tried to address most of them.

First of all, it is important to take into account that the number of apoptotic cells reported is the number of Dcp-1 positive cells the exact moment when the brain was dissected, not the accumulation of apoptotic cells until 7 days of age. But indeed, we are focusing on subtle differences between control and Appl-/- flies. As is well documented in the literature, most phenotypes in Appl-/- flies have always been very subtle. However, the fact that knocking down the expression of APPL gave similar results, difference, between control and Appl RNAi flies, shown in Fig.1 strengthens our findings. In addition, the fact that re-expressing the flAPPL and only the secreted form of APPL reduces the number of apoptotic cells to control levels is another evidence highlighting the requirement of APPL for neuronal survival.

Figure 3i has been modified and is now found in Fig.5a.

Reviewer 1 also mentions one of the key questions that arise from this work. The fact that this early increase in apoptotic cells could be due to the endolysosomal network dysregulation and or to a defective glial clearance of the accumulated neurons during the first week of life. We think that it might be due to a combination of dysregulated endolysosomal network in both neurons and glia and to a reduced glial activity (Fig.9) that leads to defective glial clearance (Fig.7-8).

About the endolysosomal network defects, we first increased the number of brains examined under live imaging and results confirmed our previous findings. Then the enlargement of endosomes under loss of APPL was also confirmed with our higher resolution, electron microscopy data. Finally, the evidence for the causal link between the enlarged early-endosomes and increase apoptotic cells at 7 days old is shown in Fig.5b where we see that reducing a copy of the early-endosome marker, Rab5, re-equilibrates the system.

**Reviewer 2:**

***General comment:***

***Kessissoglou et al. provide compelling evidence for a role of the fly homologue of APP in brain homeostasis and neuron-glia cross-talk.*** *They show that loss of APPL results in reduced lifespan, increased neuronal cell death, enlarged early-endosomal size in neurons, and reduced endolysomal size in glia. By expressing a double-tagged APPL construct in neurons of APPL -/- flies, they determine that secreted APPL is taken up by glia. Finally, expression of sAPPL in neurons of APPL -/- flies rescues glial-mediated clearance of axonal debri in an antennal ablation model.* ***These findings are of high interest for its contribution to understanding APP biology and its potential implications for Alzheimer's disease. The role of sAPP in neuron-glia cross-talk is highly novel and will be of particular interest to the field.*** *Therefore, I find this study by Kessissoglou et al to be appropriate for publication in PLOS Biology, if the following minor points can be addressed.*

We thank the reviewer for his/her positive comments on the conceptual advance this work represents, and on the manuscript itself, and for raising important issues that need to be addressed. We have performed new experiments and modified the text to address these concerns as detailed below.

***Specific comments:***

*1)	Statistics are needed in Figure S4. The difference in the survival curves between control and Appl is striking in Figure 1A. However, this difference does not seem to be replicated in Figure S4. What is the explanation for this? Overall, it is difficult to conclude from these data if there is rescue of lifespan with loss of Rab5 because the effect of APP -/- is not obvious.*

**R2.1** We agree with reviewer 2, the lifespan curve in Figure S4 does not replicate the results of Figure 1A and the early onset increase death rate rescue with one reduced copy of Rab5 was not significant. The main reason for this we think is the fact that the n number in this experiment is around 50 flies in comparison to 150 in Figure 1A. Therefore, as we did not have enough time to repeat this lifespan experiment, we decided to delete this graph and all conclusions resulting from it.

*2)	In Figure 2D, the n should be increased to at least 3 brains/genotype. In Figure 2 F-G and 4D-E, it is unclear how many brains/genotype were analyzed.*

**R2.2** We repeated the live imaging experiment and the n number in Figure 2d was increased to 5 brains/genotype. We are sorry for omitting to put the n number in Figures 2f-g and 4d-e (which is now figure 6d-e) but it has now been added in the figure legends.

*3)	Including a plot of the relative frequency for the data shown in 2D would be helpful to interpret the differences between the genotypes (similar to what was done for data in 2F and 4E).*

**R2.3** Such a plot of the relative frequency has been added in Fig.S3b.

*4)	It is unclear why there would be volumes = 0 um plotted in Figures 2D,2F,2G and 4D,E. Were all cells counted and those with no compartments were quantified as 0? If so, perhaps a better way to plot it would be to plot the % of cells with endolysosomal compartments, and then separately plot the volume for those >0. Alternatively, a size cut-off for what is considered an endolysosomal compartment may be needed.*

**R2.4** All cells were counted but only the volumes of the endolysosomal compartments are reported in this graph. Therefore, what seems to be marked as 0 is not 0um^3^ but a volume very close to 0, such as 0.01-0.1. We now provide a graph showing the distribution in a clearer way in Fig.S3e.

*5)	In Figure 3i, It seems the background of the Dcp-1 staining is higher in control than Appl -/-. Could the effect observed be an artifact? Do you have representative images with similar background staining in which this difference in Dcp-1 positive cells is still observed?*

**R2.5** This is not an artifact from the background because the settings of the microscope during imaging and the background was the same. We had shown an image in the controls of higher intensity in post processing just to emphasize the absence of signal in controls. Now Figure 3i has been modified and is the raw image without any modification of the intensity. Figure 3i has become Figure 5a. The difference in the number of DCP-1+ cells is quite evident.

*6) The manuscript is very well-written. Only a few, very minor suggested edits to the text:*

*a)	In Figure legend 1, should ***p=0.0078 be instead **p=0.0078?*

*b)	In Figure 3H, the value for *p should be added.*

*c)	A scale bar is needed in Figure 2E.*

*d)	The order of figures S5 and S6 should be switched, since S6 comes before S5 in the text.*

*e)	There is no reference to Figures S2a-b in the text.*

*f)	In the text (lines 180-191) it reads that in both Figure 4a and Figure S6 APPL was expressed in the retina; however, from the figure legends it seems that while APPL was expressed in the retina in Figure 4 , APPL was expressed in the optic lobe in Figure S6 using a different Gal4 driver- please clarify appropriately in the text.*

*g)	I'd suggest changing "strange shape" to "abnormal" or "irregular" shape in Figure 4 Legend.*

**R2.6** We have modified the manuscript and figure legends accordingly.

**Reviewer 3:**

***General comment:***

***The manuscript represents an important contribution to molecular understanding of the fly APP homologue, Appl.*** *Because the fly Appl has homology to human APP, these findings may be relevant to Alzheimer's disease.* ***Overall the manuscript is well written, and combines data from genetic manipulations with immunohistochemistry and transmission electron microscopy.*** *To test the potential implication of Drosophila Appl in the endolysosomal pathway and study its functions as signaling molecule, the authors used a loss of function Drosophila mutant in the gene. They found a small increase in cell death in the brain of 7d animals. They examined the endolysosomal organelles and found that these were larger in the appl mutant neurons. They also report interactions with rab5 and rab7. By tagging a transgene of appl with GFP on the inside and mCherry on the outside, they show that the mCherry appears in glia. They then functionally show that the ability of axonal fragments
generated upon neural body ablation (removal of the antennal lobes) is delayed in appl mutants. Adding Appl back to neurons rescues this effect. The authors suggest a model where Appl and its cleaved forms maintain the physiological flow of vesicular trafficking through the endolysosomal network in neurons and acts as signaling molecule to enable glial cells to remove the neuronal debris in the case of axotomy.*
***Overall these are interesting data that will be of interest to the field. They suggest an interesting role of appl, and an interplay between neurons and glia in Wallerian degeneration that will be of interest.***
*A number of issues, however, need to be addressed to make the results solid and compelling. The biggest concern regarding these data is that the effect is rather subtle and so the controls must be very rigorous to rule out that the effects they are seeing are simply due to genetic background.  A number of comments address this.*

We thank the reviewer for his/her positive comments on the conceptual advance this work represents, and on the manuscript itself, and for raising important experimental issues that need to be addressed. We have performed a large number of new experiments and modified the text to address these concerns as detailed below.

***Specific comments:***

*1) The authors show that the effect on cell death number in the brain is replicated by an Appl RNAi line. The line needs to be validated that it reduces Appl, and all of the other assays should also be completed with this independent approach to reducing Appl for rigor. Alternatively, they could use multiple alleles of Appl.*

**R3.1** We agree with reviewer 3 and have added a figure (Fig.S1) showing the data from two different Appl RNAi lines. This data shows that the RNAi line that reduces the most the expression of APPL is the one with the biggest effect in terms of difference in number of apoptotic cells at 7 days old compared to control. Therefore, we think that this experiment validates the Appl RNAi line that was used and presented in Figure 1d.

Unfortunately, it was not feasible to repeat all other assays reported in this paper with the Appl RNAi line but we did repeat the TEM experiment looking at the size and number of early-endosomes using the Appl RNAi line that reduced the most the expression of APPL. This is reported in the new figure Fig.S5e-g.

*2) Related to this, in Fig.1c the average of apoptotic cells (DCP1 positive cells) in 7d Control was 2 and in Appl -/- flies of the same age was 7. Whereas on Fig. S1 the average of DCP1 positive cells in 7d Control was 5 and in Appl -/- flies of the same age was still 7. Considering the discrepancies, it is not clear how robust the difference between Control and mutant is, and if it is enough to reduce the lifespan and induce neurodegeneration.*

**R3.2** We understand the concern of reviewer 3 but it should be taken into account that the number of apoptotic cells reported is the number of Dcp-1 positive cells the exact moment when the brain was dissected, not the accumulation of apoptotic cells until 7 days of age. Indeed, we are looking at a very subtle difference/phenotype and it is important to note that most APPL-/- phenotypes reported in the literature are subtle. Moreover, unfortunately, the genetic background of Appl null flies is unstable and hence every time we repeat the same experiment, we get slightly different results. This is why we often cantonized their genetic background. Moreover, the fact that knocking down the expression of APPL gave similar results, difference, between control and Appl RNAi flies, shown in Fig.1 strengthens our findings. In addition, we hope that the fact that re-expressing the flAPPL and only the secreted form of APPL reduces the number of apoptotic cells to control levels, is another evidence that makes our findings more robust. *3) The authors demonstrate strong glial disruptions and reduced volume of endolysosomal compartments in appl null flies. The authors should re-express APPL and SAPPL and rescue these phenotypes in the appl null background, to show that the effects are due to appl and not genetic background.*

**R3.3** We had already shown that re-expressing the flAPPL or just the SAPPL can rescue the defective glial clearance of degenerating axons (Fig.8). Now we performed the rescue experiment of the endolysosomal compartments phenotype and, as can observed in Figure 4b. re-expressing the flAPPL rescues the abnormal enlargement of early-endosome like vesicles. In Figure 4c. we observe that re-expressing the flAPPL or just the SAPPL rescue the increased percentage of cells containing early-endosome like vesicles. *4) For the quantification of the volume of endolysosomal compartments (Fig.2d) only 2 brains/genotype were used for analysis. It is standard to use at least 3 biological specimens and to reproduce all experiments three times or more independently.  Along the same lines, it is not clear how many biological replicates were used in experiments listed on Fig.3c, Fig. 4d, Fig. 5e, f, Fig.6b,c,d.*

**R3.4** We repeated the live imaging experiment, and the n number in Figure 2d was increased to 5 brains per genotype. We are sorry for omitting to report the n number in Figures 3c., 4d (which is now figure 6d-e), 5e,f (which is now 7e,f) and 6b-d (which is now 8b-d) but it has now been added in the figure legends. The experiments on Figures 3 and 6 consist of 5-6 biological specimens and have now been repeated twice. Because of the difficulty of performing live imaging which had to be done in collaboration with Robin Hiesinger’s lab, we could not repeat this experiment a third time due to Covid-19 confinemnet and travel restrictions. The experiments in Figure 7 and 8 have been repeated at least 3 times independently and every data point presented on the graph is the result of a brain, therefore there are more than 10 biological specimens per genotype.

*5) The rab5 and rab7 interactions should be shown with 2 different alleles as well, to rule out background effects.*

**R3.5** We are sorry but we did not have the time to address this point and perform this experiment.  *6) Does dT-APPL rescue the mutant, is this form of the protein functional?  This should be addressed.*

**R3.6** We have addressed this concern of reviewer 3 by expressing the dT-APPL in an APPL null fly and examining the mushroom body phenotype of adult flies reported by Soldano et al. 2013. In Figure S6d-e we present the data showing that dT-APPL is functional as it rescues the developmental axonal defect seen in Appl-/- flies.

*7) What happens if they just express the APPL in the glia in the axotomy model?  Potentially this may rescue and would confirm a role in glia.*

**R3.7** As far as we know until now there has been no convincing evidence that APPL is expressed in glia. Looking at the ‘’Single-cell transcriptome atlas of the aging Drosophila brain’’ by Davie et al., 2018 we confirm that APPL is only expressed in neurons and not in glia. Therefore, we considered that it is not necessary to express APPL in glia in the axotomy model because it would not represent an endogenous effect under normal conditions. The role in glia we believe is via an interaction of the secreted form of APPL which we show interacts or is taken up by glial cells. Furthermore, our results in Figure 8 present evidence that just by re-expressing the SAPPL in the olfactory neurons of an APPL-/- fly it is enough to rescue the glial clearance defect. Finally, we present new results in Figure 9 that Appl mutant flies show low expression of the Drapper protein in glia, even though glial invasion into the injury site appears normal.

*8) Other comments: Sometimes the authors mention protein forms without explaining them (sAPPL).
They use SAPPL, sAPPL and APPLS.  Which is it? Are those all refer to the same form of the protein?*

**R3.8** We agree with reviewer 3 and this has been corrected in the manuscript. They all refer to the secreted form of APPL and we changed all to SAPPL.
