# Supplementary material for: The Drosophila amyloid precursor protein homologue mediates neuronal survival and neuroglial interactions
Source: PLoS Biol. 2020 Dec 8;18(12):e3000703. doi: 10.1371/journal.pbio.3000703 (PMC7723294; doi:10.1371/journal.pbio.3000703)
Supplement: Attachment — Submitted filename: Kessissoglou et al_final_reviewer repsonse.docx [file pbio.3000703.s013.docx]

**Reviewer 1**:

*The authors addressed most of my concerns. I have two comments:*

*1. Figure legend must be added to Figure 10*

**R1.1** The figure legend has been added.

*2. On the bottom panel of Figure 10 the authors suggest that overexpression of APPLoe leads to glial overactivation and clearance of healthy neurons, which is not consistent with their results described in line 111:  "…In contrast, overexpressing SAPPL in a control background caused the presence of significantly more apoptotic cells in the brain of 7 days old flies (Figure 1f).", which means that it leads to more dying neurons and not to clearance of healthy ones. It should be changed accordingly.*

**R1.2** We agree with Reviewer 1 that this bottom panel is controversial with the results observed in Figure 1f. Therefore, we decided to remove this panel from our working model on Fig 10.

**Reviewer 2:**

*The authors have appropriately addressed my critiques, and I find this manuscript suitable for publication in PLOS Biology.*

**Reviewer 3:**

*The authors have performed considerable revisions that have increased the rigor of their data, and done a lot to make the data far more compelling.   It is an interesting story, and an important finding. The interaction with draper, to increase its protein expression and trigger phagocytosis is interesting.*

*There are a number of minor issues to be addressed/resolved.*

*1. line 21: in the abstract they say that loss of appl causes dysregulation of endolysosomal function, in both neurons and glia.  This is somewhat confusing, as they well document the defect in neurons, but the impact on glia they argue must be non-autonomous. Glia could be removed from this sentence for clarity.*

**R3.1** Although we show in Fig6c-c’ that loss of APPL causes dysregulation of the endolysosomal network also in glia, it is true that the mechanism behind APPL’s regulation of the endolysosomal network may not be the same as in neurons. Therefore, as advised by reviewer 3, for clarity, we removed ‘’glia’’ from the sentence.

*2. line187: They refer to rab5 immunostaining as "significantly broader".  It is not clear what that means - in more cells than it normally is?  or do they mean the level is higher?  this should be more clear and quantitated.*

**R3.2** As what we measured in Fig5c-d was the volume of Rab5 covered region after having set a certain fluorescence intensity threshold, the data reveal that in Appld flies we have more volume with high intensity (levels) of Rab5. This could be caused either by more early endosomes per cell or more Rab5 protein per early endosome because of their enlargement, which we have seen and reported in Appld flies. We hence modified the text and wrote ‘’significantly higher levels of Rab5’’. The quantification of is presented in Figure 5c.

*3. line 218: in figure S6e, could they point out the MB loss.*

**R3.3** The figure S6e has now been modified to mark MB β lobe loss.

*4. The addition of TEM data is nice, but in some cases it needs quantitation and they need to clarify how many animals they looked at.  For example, in figure 6g, how many times was this seen? To rule out an artifact like bad fixation.*

**R3.4** In Fig6f,g it is indeed an occasional observation that we wanted to report. We examined 3-5 animals per genotype. However, as nature, severity and penetrance of the glial shape “irregularity” were highly variable, we are not sure how best to quantify it. Therefore, we decided to move these two TEM images to Supplementary figures (S7f,gFig) and make it clear in the text that this was an occasional observation (line 237) because we still think it is an interesting observation to report. All other figures containing TEM data have been quantified and the *n* number was reported in the legend.

*5. In figure 10, they add a final panel bottom whereby they indicate that upregulation of APPL induces over activation of glia and clearance of healthy neurons.  This part of the model is likely incorrect, given they are driving expression of APPL in a rescue setting by the GAL4-UAS system at levels that are undoubtedly much higher than normal levels and yet they see only rescue. This should probably be removed or a legend should be added that this is speculative. Or they could do an experiment, with the ORNs showing active removal of neurons when up regulating APPL and upregulation of draper on all the glia. The data they have on an up regulation effect is Figure 1f, where they suggest that up regulation of sAPPL causes more cell death in the brain (mimicking the loss of function).*

**R3.5** We agree with Reviewer 3 that this bottom panel is controversial with the results observed in Figure 1f. Therefore, we decided to remove this panel from our working model on Fig 10.
